# Data on Sea turtle relative abundance in nearshore waters adjacent to the Mississippi River delta, Gulf of Mexico, United States

**DOI:** 10.1016/j.dib.2023.108984

**Published:** 2023-02-14

**Authors:** Ryan C. Welsh, Blair E. Witherington, Jeffrey R. Guertin, Cody R. Mott, Michael J. Bresette

**Affiliations:** Inwater Research Group. 4160 NE Hyline Dr. Jensen Beach, Florida, USA

**Keywords:** Vessel survey, Endangered, Protected, Louisiana, Standardized transect, Sighting data

## Abstract

We measured the relative abundance of sea turtles using standardized transect surveys conducted during the summer and fall of 2013 in neritic waters surrounding the Mississippi River delta in Louisiana, USA. Data comprise sea turtle locations, observation circumstances, and environmental covariates recorded at the beginning of each transect and at the time of each turtle observation. Turtles were recorded by species and size class, as well as location in the water column and the distance the turtle was from the transect line. Transects were performed on an 8.2 meter vessel with two observers atop a 4.5 meter elevated platform, with vessel speed standardized at ∼15 km/hr. These data are the first to describe relative abundance of sea turtles observed from small vessels in this region. Detection of turtles <45 cm SSCL and data detail are greater than aerial surveys. The data serve to inform resource managers and researchers regarding these protected marine species.

**Specifications Table**

**Table:** 

Subject	Marine Biology

Specific subject area	Vessel based sea turtle surveys
Type of data	Table Map Figure
How the data were acquired	We recorded sea turtle observations made from a small (8.2 m) vessel on standardized transect surveys undertaken in the summer and fall of 2013 in neritic waters east and west of the Mississippi River delta off Louisiana, USA. Sixteen, 50-km transects were each surveyed three times throughout the research period. Vessel transect speeds were standardized at 15 km/h. During each survey, two observers stood atop an elevated tower (eye-level = 4.5 m) positioned centrally. Turtle sightings and associated data were recorded by a separate individual in real time.
Data format	Raw
Description of data collection	Data were collected during daylight hours between the months of June and October in 2013. Surveys were only conducted when sea state allowed turtles to be readily observed near the transect line (wind speeds >15 mph). Transects were placed to represent nearshore waters and depths less than 4 m.
Data source location	• Region: Louisiana, Northern Gulf of Mexico • Country: United States of America • Latitude and Longitude: approximately 29N, −89 W
Data accessibility	Dryad Data identification number: https://doi.org/10.5061/dryad.mkkwh713w Direct URL to data: https://doi.org/10.5061/dryad.mkkwh713w

## Value of the Data

• These relative abundance data represent a spatial distribution of all sea turtle life stages over a broad area of neritic waters. Unlike aerial surveys, which also record sea turtles over large areas, these small-vessel surveys have the capability to record juveniles less than 45 cm Straight Carapace Length (SCL) (Schroeder et al. [1]).
• These are the first data representing all sea turtle life stages distributed in neritic waters adjacent to the Mississippi River delta. The data would be useful for natural resource managers who make decisions on human activities that could impact sea turtles, which are protected and endangered species. The data also serve as a baseline for future trends assessments.
• The data provide information to guide placement of transect lines for future studies.

## 1 Objective

Our objective was to represent the relative abundance and spatial distribution of sea turtles in neritic waters of Louisiana adjacent to the Mississippi Delta. The need for data like these was revealed following the 2010 Deepwater Horizon oil spill. Lack of sea turtle abundance and distribution data made damage assessment challenging and continues to hamper measurement of post-spill repopulation recovery. These data would provide baseline estimates that would apply to assessment of future catastrophic events in the same region.

## 2 Data Description

**Fig. 1:**
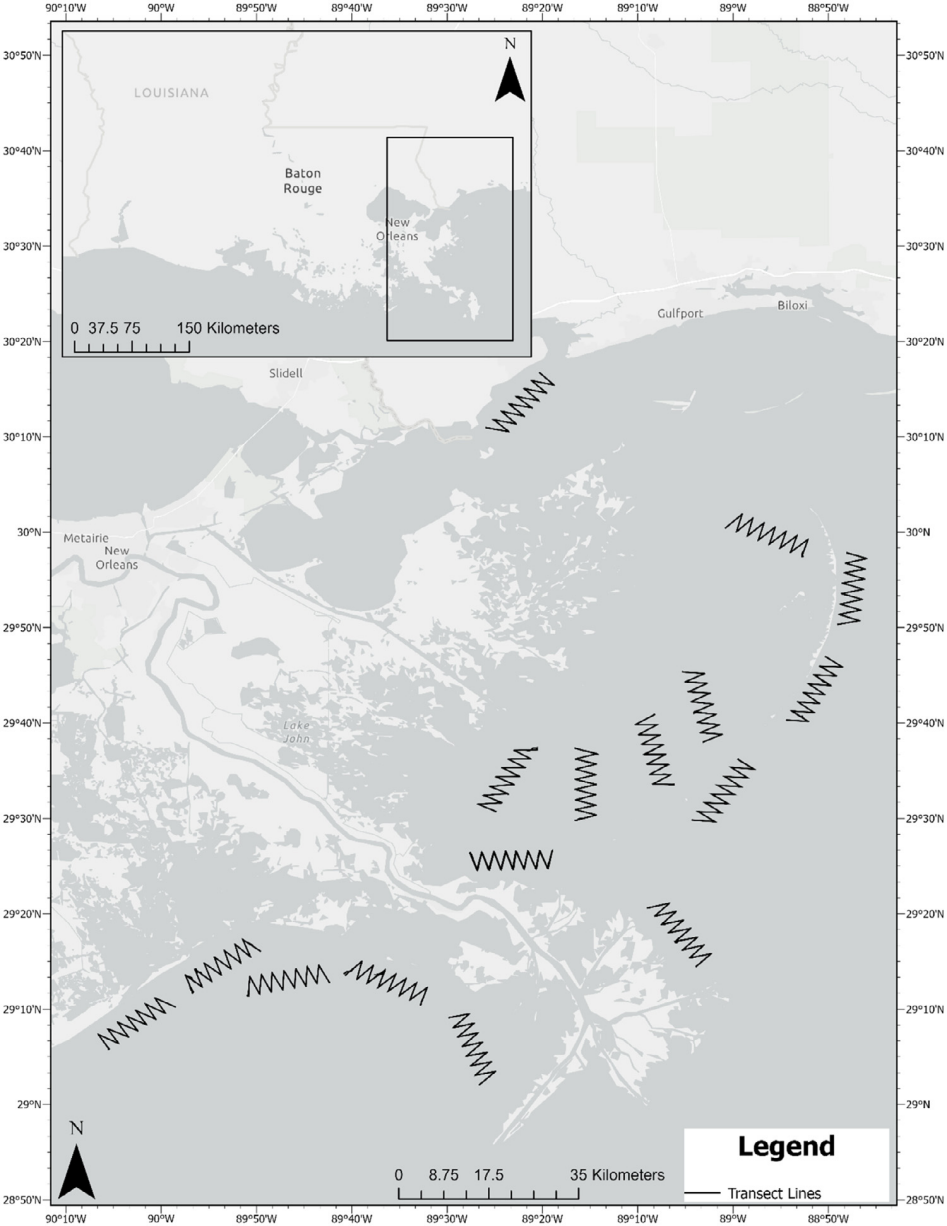
Sixteen standardized transects located in nearshore Louisiana (USA) waters adjacent to the Mississippi River delta. Each transect was run three times between the months of June and October of 2013. All start and end GPS points and tracks of transects are available at (https://doi.org/10.5061/dryad.mkkwh713w).

These data were collected to represent observations of sea turtles and associated covariates to allow assessment of detectability and estimation of relative abundance. Observations were made during 48, vessel-based surveys, each ∼50 km in length. The surveys comprised 16 predetermined, zigzag transect lines that represented nearshore Louisiana waters <4 m depth, adjacent to the Mississippi River delta (Fig. 1). Each of the 16 zigzag transects had an approximate orientation parallel to the adjacent coastline. Five transects were west of the delta, within three miles of shore between Port Fourchon, Louisiana and the Southwest pass of the Mississippi River delta. The remaining 11 transects were east of the Mississippi River delta, between South Pass of the Mississippi River delta and Heron Bay in western Mississippi.

Environmental conditions during transects recorded an average wind speed of 6.5 mph (range: 0 – 15 mph), average wave height of 2.3 ft (range: 1 – 4 ft) and average water clarity of 1.5 m (range: 1 – 3 m).

During these transects, we recorded 48 observations with a maximum distance of sighting from the transect line at 150 m. The species composition of these sightings are as follows: 20 loggerhead sea surtles (*Caretta caretta*), 20 Kemp's ridleys (*Lepidochelys kempii*), 7 leatherbacks (*Dermochelys coriacea*), and 1 green turtle (*Chelonia mydas*). Size classes for each species were: loggerheads (13 adults, 7 juveniles), Kemp's ridleys (6 adults, 14 juveniles), leatherbacks (4 adults, 3 juveniles) and 1 juvenile green turtle. Of the 48 sightings, only 7 (3 loggerheads and 4 Kemp's ridleys) were observed below the surface of the water (14.5%).

**Fig. 2:**
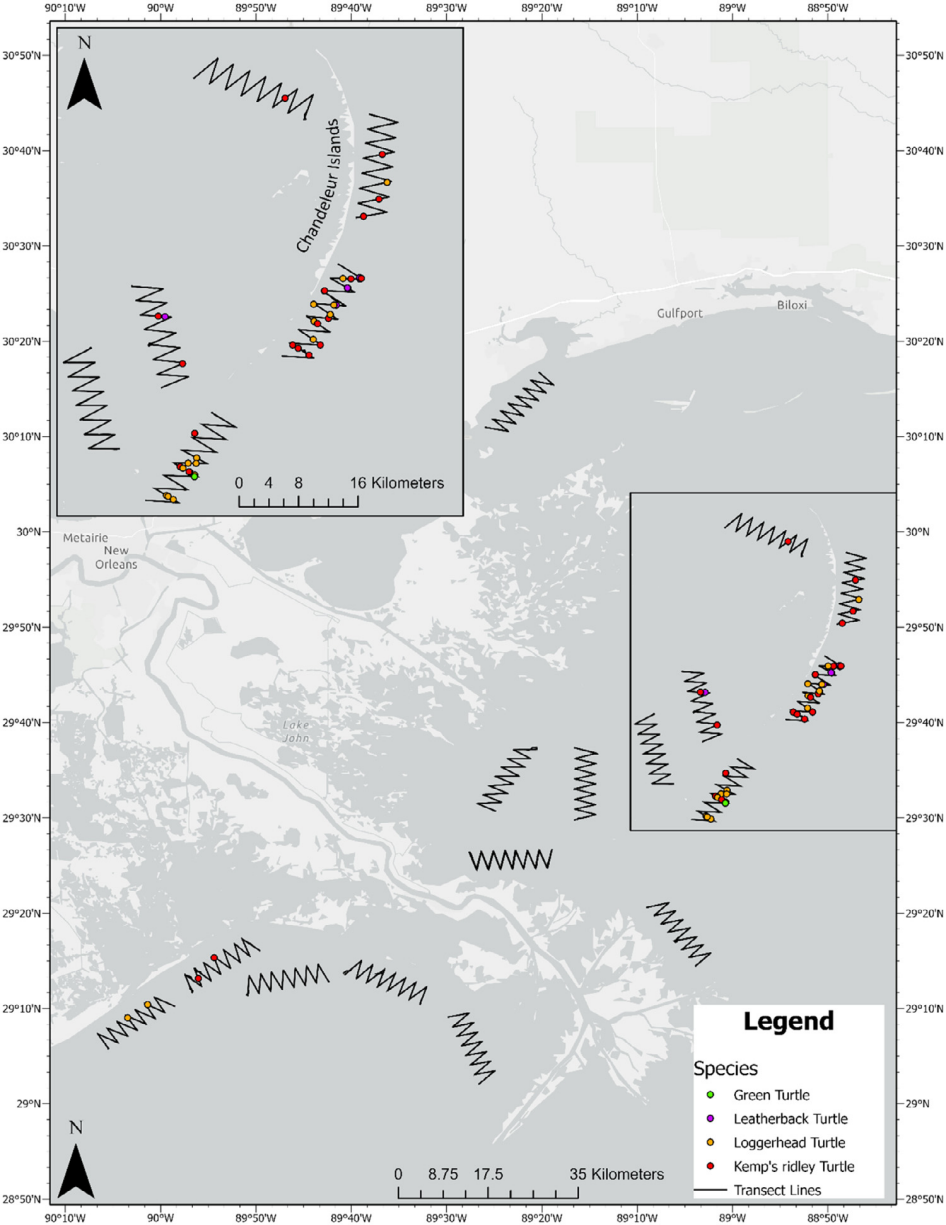
Sixteen standardized transects and all turtle observations located in nearshore Louisiana (USA) waters adjacent to the Mississippi River delta. Each transect was run three times between the months of June and October of 2013. Inset map is a close-up of the waters around the Chandeleur Islands where 91% of all marine turtle observations occurred. All start, end, and observation GPS points and tracks of transects are available at (https://doi.org/10.5061/dryad.mkkwh713w).

Although 5 of the 16 transects (31.2%) were west of the Mississippi River delta, only 4 (8.3%) of the observations (2 loggerhead, 2 Kemp's ridleys) occurred in that region. The majority (91.7%) of marine turtle observations (18 loggerheads, 18 Kemp's ridley, 7 leatherbacks, and 1 green turtle) occurred to the east of the Mississippi River delta. Within the area east of the Mississippi River delta, all observations were along 5 transects surrounding the Chandeleur Islands, which are a barrier island chain encompassed by the Breton Island National Wildlife Refuge (Fig. 2).

## 3 Experimental Design, Materials and Methods

Each transect was surveyed at approximately 15 km/hr during three non-consecutive survey days between the months of June and October in 2013. Transect and recording methods are described in Welsh and Mansfield [2]. We used two observers atop an elevated platform (eye-level = 4.5 m) on an 8.2 m survey vessel. While underway, a helmsman ensured the survey vessel stayed on course and recorded vessel path and turtle locations with a Garmin Global Positioning System unit. A separate individual recorded data in real time from the observers.

Environmental data, recorded prior to the start of each transect, included wave height, cloud cover, water temperature, water clarity, wind speed and wind direction. For each turtle observation, we recorded species, size class, position in the water column, and perpendicular distance from the transect line. Observers categorized all sighted turtles by species and size class, broadly classified as either adult or juvenile. Observers determined size class by using relative size of the carapace in terms of Straight Carapace Length (SCL) as a guide. Measurements delineating the different size classes (adults, juveniles) were defined using recommendations by the Florida Fish and Wildlife Conservation Commission Marine Turtle Conservation Handbook [3] and Eaton et al. [4]. Delineation of adult and juvenile size classes are as follows: loggerhead and green turtles: Adults (≥85 cm SCL), Juveniles (<85 cm SCL); Kemp's ridley: Adults (≥60 cm SCL), Juveniles (<60 cm SCL); leatherbacks: Adults (≥135 cm SCL), Juveniles (<135 cm SCL).

## Ethics Statements

These data are based on purely observational data collection and involved no contact or manipulation of animals. As such, the study complies with all wild animal, protected and endangered species ethics guidelines.

## CRediT authorship contribution statement

**Ryan C. Welsh:** Formal analysis, Data curation, Writing – original draft, Investigation, Visualization. **Blair E. Witherington:** Conceptualization, Methodology, Supervision, Investigation, Writing – review & editing. **Jeffrey R. Guertin:** Investigation, Writing – review & editing. **Cody R. Mott:** Investigation. **Michael J. Bresette:** Conceptualization, Methodology, Supervision, Investigation, Project administration, Funding acquisition, Writing – review & editing.

## Declaration of Competing Interest

The authors declare that they have no known competing financial interests or personal relationships that could have appeared to influence the work reported in this paper.

## Data Availability

Sea turtle relative abundance in nearshore waters adjacent to the Mississippi River delta, Gulf of Mexico, United States (Original data) (Dryad).
